# Potential Mechanisms Underlying the Deleterious Effects of Synthetic Cannabinoids Found in Spice/K2 Products

**DOI:** 10.3390/brainsci9010014

**Published:** 2019-01-16

**Authors:** Balapal S. Basavarajappa, Shivakumar Subbanna

**Affiliations:** 1 Division of Analytical Psychopharmacology, Nathan Kline Institute for Psychiatric Research, Orangeburg, NY 10962, USA; Subbanna.Shivakumar@nki.rfmh.org; 2 New York State Psychiatric Institute, New York, NY 10032, USA; 3 Department of Psychiatry, College of Physicians& Surgeons, Columbia University, New York, NY 10032, USA; 4 Department of Psychiatry, New York University Langone Medical Center, New York, NY 10016, USA

**Keywords:** marijuana, cannabinoid receptors, K2, Spice, gene expression, CREB

## Abstract

The chief psychoactive constituent of many bioactive phytocannabinoids (Δ^9^-tetrahydrocannabinol, Δ^9^-THC) found in hemp, cannabis or marijuana plants are scientifically denoted by the Latin term, *Cannabis sativa*, acts on cell surface receptors. These receptors are ubiquitously expressed. To date, two cannabinoid receptors have been cloned and characterized. Cannabinoid receptor type 1 (CB1R) is found to serve as the archetype for cannabinoid action in the brain. They have attracted wide interest as the mediator of all psychoactive properties of exogenous and endogenous cannabinoids and they are abundantly expressed on most inhibitory and excitatory neurons. Recent evidence established that cannabinoid receptor type 2 (CB2R) is also expressed in the neurons at both presynaptic and postsynaptic terminals and are involved in neuropsychiatric effects. Distinct types of cells in many regions in the brain express CB2Rs and the cellular origin of CB2Rs that induce specific behavioral effects are emerging. To mimic the bliss effects of marijuana, synthetic cannabinoids (SCBs) have been sprayed onto plant material, and this plant material has been consequently packaged and sold under brand name “Spice” or “K2”. These SCBs have been shown to maintain their affinity and functional activity for CB1R and CB2R and have been shown to cause severe harmful effects when compared to the effects of Δ^9^-THC. The present review discusses the potential brain mechanisms that are involved in the deleterious effects of SCBs.

## Introduction

**Figure 1:**
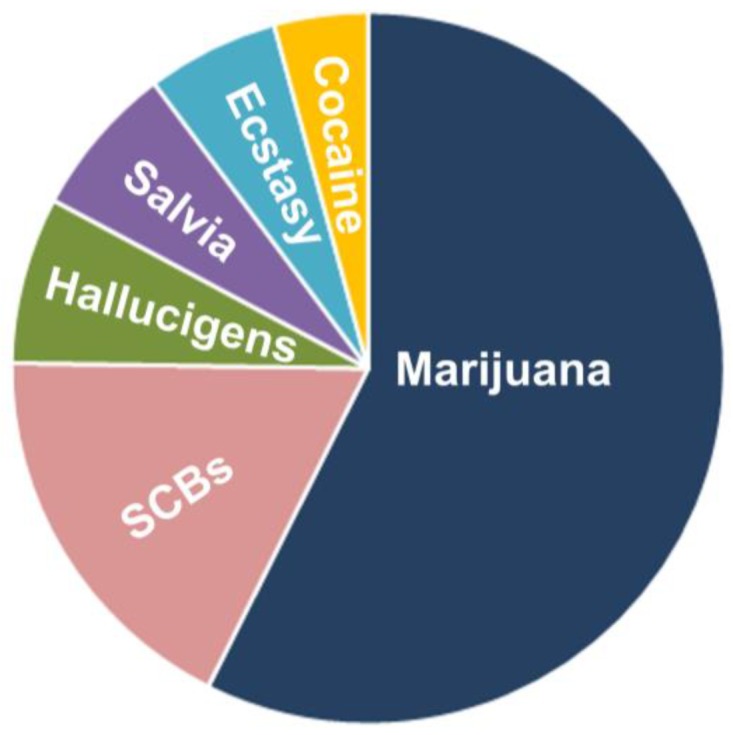
Pie illustration outlining the synthetic cannabinoid (SCB) abuse rate among high school-going children. SCB abuse is the most widespread among young people; of the illicit drugs most used by high school seniors, the use of SCBs are second only to that of marijuana (http://www.drugabuse.gov/publications/drugfacts/spice-synthetic-marijuana). The most common reasons for using SCBs were affordability, inability to detect SCBs in standard drug tests, and perceived physical and emotional benefits.

Marijuana is the major often abused illegal drug (Figure 1). The degree of abuse raised from 14.5 million in 2007 to 18.1 million in 2011, with a projected about 5 million adult daily cannabis abusers. Marijuana abuse appears to begin in the eighth grade in 1.3% of children and between 12 and 17 years old in 7.9% of children [1]. Besides, the typical content of Δ^9^-tetrahydrocannabinol (Δ^9^-THC), which is a major psychoactive component of many bioactive cannabinoids established in the *Cannabis sativa* plant [2], in marijuana has also doubled from 3.4% in 1993 to 8.8% in 2008 [3]. Moreover, an upsurge in the concentration of Δ^9^-THC (13.8%) in highly potent plant varieties (sinsemilla, ‘skunk’) [3] was also found in this period. The psychological effects that were detected after Δ^9^-THC use are comparable to those observed after recreationally consumed cannabis [4]. The majority of Δ^9^-THC effects are exerted via the endocannabinoid (EC) system. The EC system comprises receptors for Δ^9^-THC known as cannabinoid receptors type 1 and 2 (CB1R and CB2R, respectively), endogenous receptor ligands (‘endocannabinoids’, ECs), and EC synthesizing and degrading enzymes [5]. CB1R is ubiquitously expressed in brain regions, such as the hippocampus, basal ganglia, cortex, amygdala, and cerebellum, all of which are areas connected with the behavioral effects of Δ^9^-THC [6]. The EC system has a homeostatic role, but its dysfunction can promote pathological conditions [7,8].

**Figure 2:**
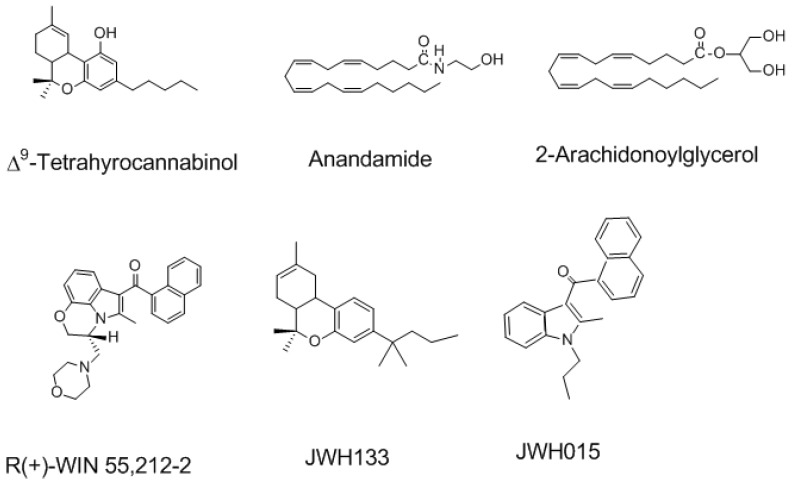
The structure of natural, endogenous and selective synthetic cannabinoid agonists of Cannabinoid receptors type 1 and type 2 (CB1R and CB2R).

**Figure 3:**
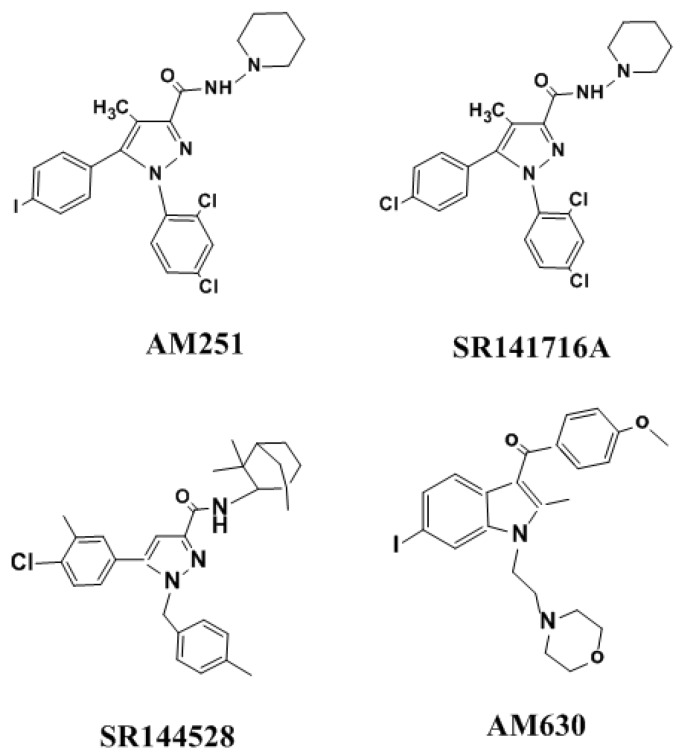
The structure of selective CB1R and CB2R antagonists.

The CB1Rs and CB2Rs belong to the large superfamily of heptahelical G protein-coupled receptors (GPCR) and couple with G_i_/_o_ proteins (for more details, see reviews [5,7]). CB1R is one of the highly abundant GPCRs in the brain, with densities that are similar to the levels of γ-aminobutyric acid (GABA)- and glutamate-gated ion channels [9]. Functional CB2R is also present in limited amounts and distinct locations in the brains of several animal species, including humans [10]. The existence of CB2R in the brain has been acknowledged in distinct locations of the CNS in many animal species, including humans, in moderate levels, and is restricted to microglia and vascular elements [11]. However, the specific functions of this receptor in the CNS are emerging slowly. Recent strong evidence suggests the presence of CB2R mRNA in neuronal cells of the hippocampus [12] and dopamine-expressing neurons in the ventral tegmental area (VTA) [13,14]. CB2R-mediated regulation of cell type-specific synaptic plasticity was shown in the hippocampus [15,16,17]. Furthermore, increased CB2R levels in neurons were noticed under pathological conditions [18,19]. The selective agonists and antagonists of CB1R and CB2R are shown in Figure 2 and Figure 3.

**Figure 4:**
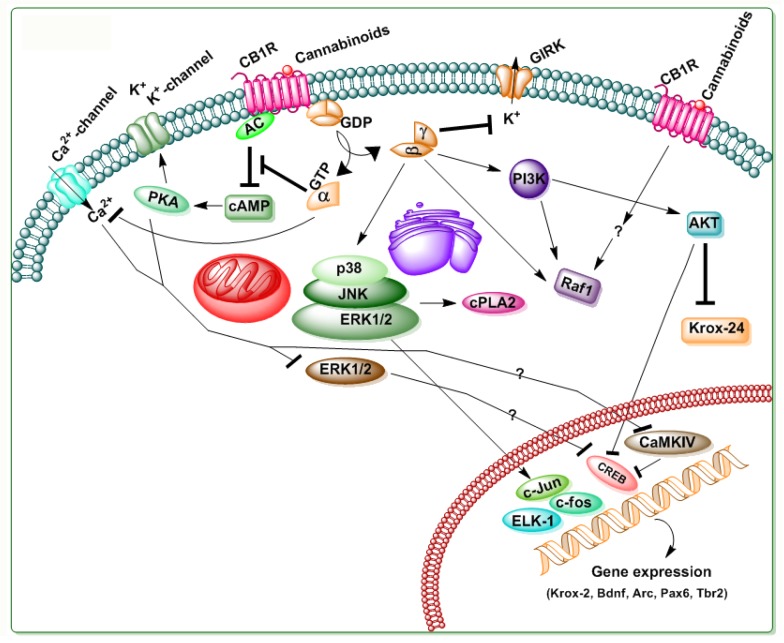
Schematic illustration of the CB1R mediated signaling. Δ^9^-tetrahydrocannabinol (Δ^9^-THC) and other SCBs produce their effects via binding to CB1Rs, a 7-transmembrane domain G-protein coupled receptor located in the cell membrane. The Ca^2+^ channels inhibited by CB1R stimulation include N-, P/Q-, and L-type channels. The actions on Ca^2+^ channels and adenylyl cyclase (AC) are thought to be mediated by the α subunits of the G-protein, and the effects on G protein-coupled inward rectifying K^+^ (GIRK) and PI3K are considered to be mediated by the βγ subunits. AC inhibition and the subsequent decrease in cyclic adenosine monophosphate (cAMP) lead to the inhibition of cAMP-dependent protein kinase (PKA). This leads to decreased K^+^ channels and pCaMIV and pCREB levels, which might lead to the inhibition of gene expression required for several physiological functions. Stimulatory effects are indicated by (→) symbols and inhibitory effects by (⊥) symbols.

The stimulation of CB1R promotes its interaction with G-proteins, resulting in guanosine diphosphate/guanosine triphosphate exchange and the subsequent dissociation of the α and βγ subunits. These subunits regulate the activity of multiple downstream effector proteins to produce biological functions. CB1Rs are coupled with G_i_ or G_o_ proteins. However, their affinity for G_i_ or G_o_ proteins might vary, as revealed by several receptor ligands and receptor ligand-stimulated GTPγS-binding studies [20]. CB1R activity differs from several other GPCR-G proteins, as it is precoupled with G-proteins and it is hence constitutively functional in the absence of exogenous agonists [21]. The CB-mediated signal transduction pathway is shown in Figure 4. Activation of CB1R by R-(+)-methanandamide (MetAEA) and ECs in N18TG2 cells inhibited adenylate cyclase (AC) activity (for review see [7]). In certain conditions, the enhanced AC activity was reported without G_i_/_o_ coupling (pertussis toxin-sensitive), probably through the stimulation of G_s_ proteins [22]. In vitro experiments, the expression of specific isoforms of AC (I, III, V, VI, or VIII) with the coexpression of CB1R was shown to inhibit cyclic adenosine monophosphate (cAMP) accumulation. However, the expression of II, IV, or VII AC isoforms, along with the CB1R coexpression was shown to enhance cAMP accumulation [23]. Interestingly, whether the coupling of CB1R with G_s_ proteins has physiological function and whether this coupling enhances after G_i_ or G_o_ protein removal through colocalized noncannabinoid G_i_/_o_ protein-coupled receptors have yet to be investigated. Further studies of the mechanism through which CB1R stimulation primes to the buildup of GαGTPβγ heterotrimers would improve our knowledge on the CB1R mediated signal transduction mechanisms. It is also imperative to establish whether these heterotrimers (Gα, Gβγ, and GαGTPβγ) can cooperate with distinct downstream effector targets to afford specificity to the signaling pathways.

**Figure 5:**
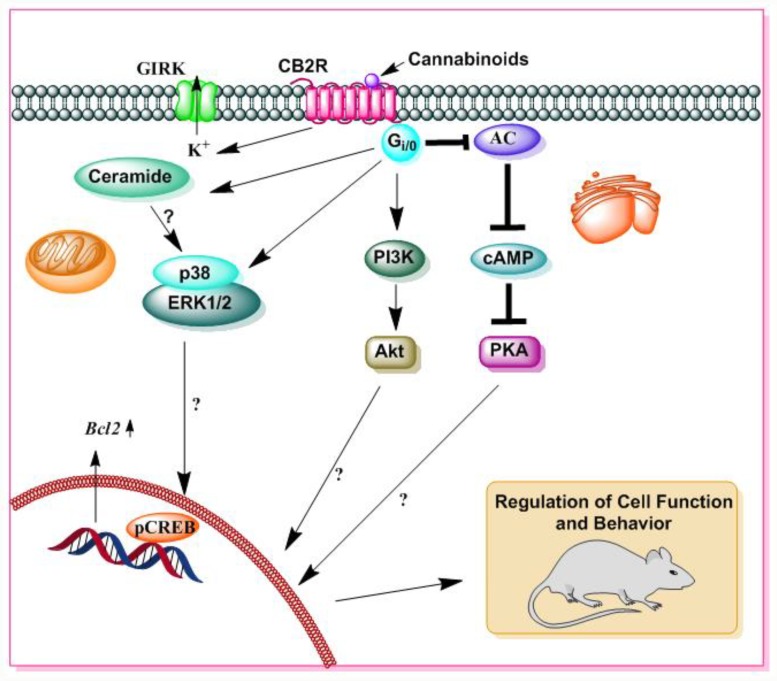
A schematic diagram of CB2R mediated signaling. Δ^9^-THC and SCBs also bind to CB2R. Similar to CB1R, CB2R is also a 7-transmembrane domain G-protein coupled receptor that is located in the cell membrane. The activation of CB2R is associated with several distinct cellular processes, such as GIRK, adenylate cyclase (AC), cAMP, PKA, ERK, p38 MAPK, and Akt pathways, as well as the pathway for the de novo synthesis of ceramide. Stimulatory effects are represented by (→) symbols and inhibitory effects by (⊥) symbols.

CB2R belongs to the seven-transmembrane domain class of GPCRs. CB2R is also coupled to G_i_/_o_ proteins; thus, the stimulation of CB2R is associated with the inhibition of AC and the cAMP/PKA-dependent pathway, as has been observed for CB1R. CB2R stimulation activates MAPK cascades, specifically the ERK and p38 MAPK cascades [24]. Additionally, the activation of CB2R has also been linked to the stimulation of additional intracellular pathways, including the PI3K/Akt pathway [24] (Figure 5). These pathways have been associated with pro-survival effects, as well as with the de novo synthesis of the sphingolipid messenger ceramide [24], which has been linked to the pro-apoptotic effects of CBs. CB2R activation by the selective agonist AM1241 contributes to the regeneration of DA neurons through increased activation of the PI3K/AKT signaling pathway following MPTP-induced neurotoxicity in mice [25]. Activation of CB2R by JWH133 induced neuroinflammation by enhancing the expression of MAPK phosphatase-1 (MKP-1), followed by the inhibition of MAPK signaling and increased blood-brain barrier permeability in a rat model of intracerebral hemorrhage (ICH) [26]. The activation of CB2R is also associated with the activation of CREB, followed by increased expression of Bcl-2, an anti-apoptotic gene leading to decreased cleaved caspase-3 levels and augmented neurological deficits induced by subarachnoid hemorrhage (SAH) in male rats [27]. The activation of CB2R has been shown to increase G protein-coupled inward rectifying K^+^ (GIRK) channel activity in pyramidal cells [28]. The activation of CB2R regulates the function of the sodium-bicarbonate cotransporter, causing a hyperpolarization of the neuron in a self-regulatory manner, independent of CB1R activity. These results provide additional evidence for the neuronal expression of CB2Rs and underscore the function of CB2R in neuronal transmission [17].

SCBs were synthesized as research tools to investigate the function of the EC system [29]. However, within the last decade, SCBs have been sprayed onto plant material, which has then been packaged and sold as “Spice” or “K2”. SBCs are also sold as liquids that can be vaporized and inhaled via e-cigarettes or other devices. These products are also identified as herbal or liquid incenses. These products mimic the effects of marijuana [30], because SCBs act on the same CB receptors as Δ^9^-THC, the psychoactive component in marijuana. Although these packages are labeled “not for human consumption”, these products are often smoked, resulting in a marijuana-like high, as well as other physiological effects. These products are often marketed as safe, legal alternatives to marijuana. They are dangerous and may affect the brain much more strongly than marijuana; their particular effects can be variable and, in some cases, fatal. Many synthetic cannabinoids (SCBs) are now classified as Schedule I drugs under the United States (U.S.) Controlled Substances Act [31]. Because SCBs are now illegal and are classified in the most dangerous category of scheduled drugs, manufacturers have attempted to circumvent these laws by changing the more structurally diverse cannabimimetic compounds in their mixtures, which might not be listed under scheduled drug regulations. In addition, “medical” marijuana is being legalized in many states across the U.S. Therefore, individuals who previously would not have risked the consequences of procuring an illegal drug might now consider exposing themselves to marijuana and SCBs. In addition, the legal availability of marijuana may decrease use of SCB in the future.

SCBs were classified (Table 1) based on the chemical structures of the molecules [32]. The pharmacological effects of many SCBs vary widely and have been recently reviewed [33]. The most frequently investigated SCBs are the aminoalkylindole WIN55,212-2, the cyclohexylphenol CP55,940, and HU-210. Only a few studies have examined the naphthoylindoles (JWH-018 and analogs) or the newer synthetic structural families of cannabinoids that are now dominating the “Spice” market [33]. SCBs and their metabolites have been found to possess a higher binding affinity for CB receptors than Δ^9^-THC, which implies greater potency, greater harmful effects, and perhaps a longer duration of action. Although most Spice cannabinoids are widely known to be potent CB1R agonists, knowledge of the exact mechanisms that underlie their severe deleterious effects is largely limited. In this review, the authors provide an integrative overview of the current research on the mechanisms that are involved in the detrimental effects of Spice compounds.

Spice products comprise several SCBs, and their potency at CB1R and CB2R differ. Therefore, their signaling mechanisms may also vary. Recently, a list of SCBs detected in Spice products, as well as the effects of these cannabinoids, was reviewed [33]. Some known physiological effects of SCBs are listed in Table 2. SCBs with a higher affinity for either CB1R or CB2R were shown to elicit adverse neurobehavioral effects. The majority of SCBs found in Spice products (Table 3), such as AM5983, AM678, AM2233, AM2389, SDB-001, AM4054, UR-144, XLR-11, JWH-081, and JWH-073, were shown to have higher affinity for CB1R than CB2R; these SCBs are expected to be similar to Δ^9^-THC in their action and suggested to be more severe than Δ^9^-THC, with some of them displaying other symptoms [34]. A few SCBs found in Spice products, such as JWH-018, AM1710, and JWH-133, shown to have a higher affinity for CB2R than for CB1R (for references, see (https://www.drugs-forum.com/forum/showthread.php?t=117873)). Selected CB1R and CB2R agonists appear to bind off target sites, such as sodium channels, μ and δ opioid receptors, muscarinic acetylcholine receptors, 5-HT_3_ receptors, and glycine receptors (for more details see [35]). Although details are limited, some designer drugs that are found in spice products have been shown to exert their action through non-CB1R and CB2R targets [36,37,38]. However, the mechanisms by which SCBs instigate their potentially harmful effects are not well established.

**Figure 6:**
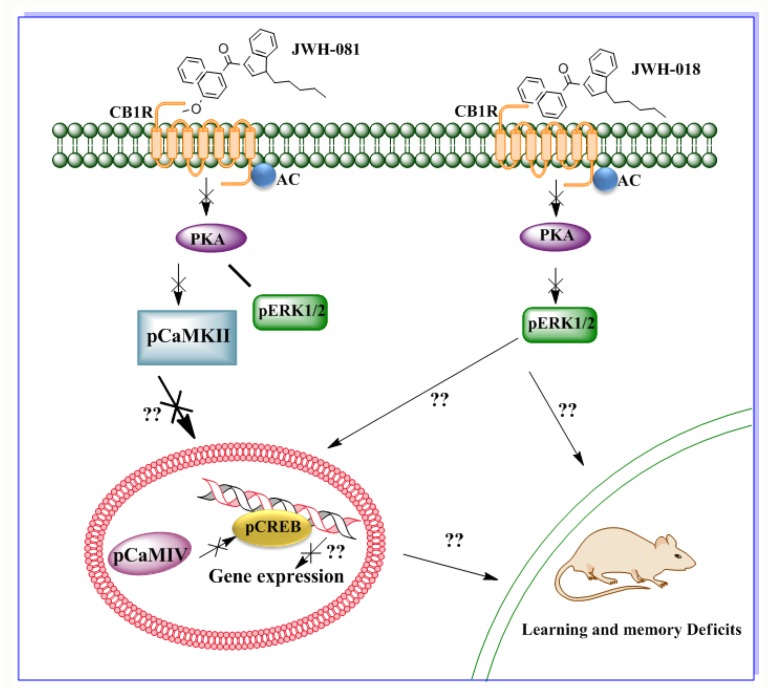
Schematic diagram showing the CB1R signaling mechanism of the SCBs found in Spice products. JWH-081 and JWH-018 both act on CB1R but activate distinct CB1R signaling events. JWH-018, which has a higher affinity for CB2R than for CB1R, reduces pERK1/2. JWH-081, which has a stronger affinity for CB1R than for CB2R, does not affect pERK1/2 but does impair pCaMKIV and pCREB levels, which are linked with Arc gene expression.

JWH-081 produces acute toxicity, as demonstrated by emergency patients, perhaps as a result of strong CB1R activation [34]. JWH-081 binds to CB1R with high affinity (1.2 nM) [74,75]. Bath application (acute) of JWH-081 to hippocampal slices impaired long-term potentiation (LTP). This effect was absent in CB1R KO mice slices. Thus, the adverse effects of JWH-081 on LTP are through CB1R activation. Acute JWH-081 administration in adult mice impairs spontaneous alternation as well as spatial memory in the Y maze and object recognition memory. The participation of pCaMKIV, pCREB, and pERK1/2, which are critical for synaptic plasticity, learning, and memory [76] in the acute effects of JWH-081 has also been demonstrated. JWH-081 impaired pCaMKIV and pCREB levels in the hippocampi of CB1R WT but not KO mice. JWH-081 decreased pCaMKIV levels in a dose-dependent manner, while the reduced pCREB was found only at a higher dose of JWH-081 (1.25 mg/kg, 30 min). JWH-081 failed to alter pERK levels and total ERK protein levels in the hippocampi of CB1R WT or KO mice. Further, preadministration of the CB1R antagonist SR141716A 30 min before JWH-081 treatment augmented both loss of pCaMKIV and pCREB levels. JWH-081 exhibited greater in vitro and in vivo responses as compared to Δ^9^-THC [77]. Overall, the CaMKIV-mediated phosphorylation of CREB at Ser133 is crucial for the transcriptional activation of the CREB/CRE-mediated gene expression [78] and it has been established to play an essential role in memory consolidation and LTP [76]. Δ^9^-THC significantly reduced the pCREB levels [79] and another calmodulin kinase-related molecules, such as pCaMKII, in a CB1R-dependent manner [80]. All Δ^9^-THC metabolites, except one, are inactivated through oxidative metabolism [81], which block further CB1R stimulation. The higher affinity, potency, and efficacy of JWH-081 [82], coupled with its likelihood to be metabolized into other metabolites [83], demonstrates that both the acute and chronic effects of JWH-081 might be more important than those of a similar dose of Δ^9^-THC. Hence, JWH-081 impairs pCaMKIV and pCREB activities through a signaling mechanism downstream of CB1R to elicit potent deleterious neurobehavioral effects in mice (Figure 6). The SCBs, JWH-018, AM2201, and XLR-11, all inhibit glutamate release and impair LTP in the mouse hippocampus in a CB1R-dependent manner [49]. JWH-018 and its halogenated derivatives (JWH-018-Cl, JWH-018-Br) dose-dependently impaired both short- and long-memory retention in mice and diminished electrically evoked synaptic transmission, LTP, glutamate, and GABA release in hippocampal slices [67].

Another derivative of JWH, JWH-018, displays agonistic activity at CB1R (9 nM) and CB2R (2.94 nM) [84] and it produces the tetrad of behaviors that are classically associated with CBs in rodent models (analgesia, catalepsy, hypomotility, and hypothermia) [39,43], but these effects are less potent than those that are associated with JWH-081. Chronic JWH-018 treatment also induces deficits in spatial memory in adolescent mice [85]. JWH-018 inhibits forskolin-stimulated cAMP production [86] and has been shown to reduce pERK levels in cultured hippocampal neurons [48]. While the effects of JWH-081 and JWH-018 are evidently due to CB1R activation, the likely function of CB2R in the effects of compounds that are present in “Spice” and “K2” warrant future investigation.

Another SCB, MAM-2201 (1-(5-fluoropentyl)-1H-indol-3-yl)(4-methyl-1-naphthalenyl)-methanone, suppressed neurotransmitter release at CB1R-expressing Purkinje cell (PC) synapses in cerebellum slices. MAM-2201 caused more significant inhibition of neurotransmitter release than Δ9-THC and JWH-018. The reduced neurotransmitter release from CB1R-containing PC synapses could contribute to some of the symptoms of SCB intoxication, including impairments in cerebellum-dependent motor coordination and motor learning [87]. Several SCBs have been shown to be cytotoxic in in vitro studies. SCBs, such as CP-47,497 and CP-47,497-C8, exhibited caspase-3- and CB1R-dependent cytotoxicity in NG 108-15 cells, suggesting that caspase cascades may have a significant function in the apoptosis induced by these SCBs [88]. XLR-11, at biologically appropriate doses (in the nanomolar range), has been shown to affect mitochondrial function in human proximal tubule (HK-2) cells through a CBR- and eCB-related mechanism. XLR-11 has been shown to induce a transient hyperpolarization of the mitochondrial membrane and enhance ATP production, followed by Bax translocation from the cytosol into the mitochondria. These events cause energy-dependent apoptotic cell death via mechanisms, such as increased caspase-3 activity and chromatin condensation [89]. Furthermore, JWH-018, JWH-073, and several major human metabolites of these compounds exhibited a high affinity for CB2Rs in CHO-hCB2 cells, suggesting that, when CB2R is available in abundance, these SCBs and their metabolites readily bind to CB2R with high affinity and elicit CB2R signaling [90]. The SCB AM2201 has been shown to induce epileptic seizures by enhancing CB1R-mediated glutamatergic transmission in the hippocampus [91].

The SCB CP-47,497-C8 was shown to induce chromosomal damage, suggesting that SCB could cause genetic instability in SCB abusers [92]. Halogenated derivatives of JWH-018 (JWH-018 Cl and JWH-018 Br) have been shown to impair motor activity and induce catalepsy in mice, and their effects were more severe than those of Δ^9^-THC. When compared to JWH-018, JWH-018Br was less effective at causing seizures, myoclonia, and hyperreflexia. These findings suggest that the halogenated compounds might have been used in the Spice products to produce similar intoxicating effects as JWH-018 while producing fewer side effects [68]. In another in vivo study, repeated administration of JWH-018 transiently enhanced 5-HT1A receptor sensitivity to produce tolerance to its hypothermic and cataleptic effects [36]. It has been shown that JWH-018 undergoes extensive metabolism by cytochrome P450 (P450). The major enzyme involved in this metabolism is CYP2C9, a highly polymorphic enzyme that is found largely in the intestines and liver. The polymorphic nature of CYP2C9 results in variable levels of biologically active JWH-018 metabolites in some individuals, offering a mechanistic explanation for the diverse clinical toxicity often observed following JWH-018 abuse [93]. Recently developed SCBs, such as MMB-FUBINACA, MDMB-FUBINACA, CUMYL-PICA, 5F-CUMYL-PICA, NNEI, and MN-18, exhibited a high affinity for human CB1R and CB2R and produced greater effects than Δ^9^-THC in [^35^S] GTPγS binding and cAMP signaling assays. Additionally, all six synthetic cannabinoids replaced Δ^9^-THC in drug discrimination, suggesting that these SCBs may possess subjective effects that are comparable to those of cannabis. Notably, MDMB-FUBINACA, a methylated analog of MMB-FUBINACA, exhibited a higher affinity for CB1R than the parent molecule, suggesting that slight structural alterations could cause a larger impact on the pharmacological properties of these drugs [65]. AM2201 and XLR-11 resulted in hypothermia in a CB1R-dependent manner in mice [94]. JWH-018 administration inhibited sensorimotor responses at lower doses, reduced spontaneous locomotion at intermediate/high doses, and induced convulsions, myoclonia and hyperreflexia at high doses in male CD-1 mice. These SCB effects were CB1R-dependent and directly implicated in SCB abuse and driving [69]. JWH-250 and JWH-073 exhibited nanomolar affinity and potency in in vitro competition binding experiments that were performed on a mouse and human CB1R and CB2R. In vivo administration of either JWH-250 or JWH-073 induced marked hypothermia and increased the pain threshold to both noxious mechanical and thermal stimuli. In addition, it also caused catalepsy, reduced motor activity, impaired sensorimotor responses (visual, acoustic and tactile), caused seizures, myoclonia, and hyperreflexia; and, promoted aggressiveness in male CD-1 mice. Furthermore, a microdialysis study in freely moving mice showed that systemic administration of JWH-250 or JWH-073 stimulated dopamine release in the nucleus accumbens. The CB1R agonist AM251 fully rescued behavioral, neurological, and neurochemical effects. Furthermore, coadministration of ineffective doses of JWH-250 and JWH-073 impaired visual sensory motor responses, augmented the mechanical pain threshold, stimulated mesolimbic DA transmission, and affected other behavioral and physiological parameters [71]. In other in vivo studies, JWH-018 and AKB48 facilitated spontaneous locomotion in mice. These behavioral effects were inhibited by CB1R and dopamine (DA) D_1_/_5_ and D_2_/_3_ receptor blockade. SPECT-CT studies with dopamine transporter (DAT) revealed that JWH-018 and AKB48 decreased [^123^I]-FP-CIT binding in the mouse striatum. Moreover, microdialysis studies showed that the in vivo administration of JWH-018 or AKB48 activated DA release in the nucleus accumbens (NAc) of freely moving mice. These results suggest that JWH-018 and AKB48 induced a psychostimulant effect in mice, possibly by increasing NAc DA release [72]. In other in vivo studies, 5F-ADBINACA, AB-FUBINACA, and STS-135 caused hypothermia; enhanced pain threshold to both noxious mechanical and thermal stimuli; induced catalepsy; impaired motor activity; resulted in deficits in sensorimotor responses (visual, acoustic, and tactile); induced seizures, myoclonia, and hyperreflexia; and, promoted aggressiveness through CB1R in mice [70]. However, the visual sensory response that is induced by STS-135 was only partially prevented by AM 251, suggesting an additional CB1R-independent mechanism [73]. Two recently developed SCBs (AKB48 and 5F-AKB48) exhibited nanomolar affinity and potency at CBRs in mouse and human cell preparations in in vitro competition binding experiments. Administration of AKB48 or 5F-AKB48 in CD-1 mice elicited hypothermia; enhanced the pain threshold to both noxious mechanical and thermal stimuli; induced catalepsy; impaired motor activity; resulted in defects in sensorimotor responses (visual, acoustic and tactile); elicited seizures, myoclonia, and hyperreflexia; and, promoted aggressiveness in mice. Moreover, a microdialysis study in freely moving mice showed that in vivo administration of AKB48 and 5F-AKB48 increased dopamine release in the nucleus accumbens. These behavioral, neurological, and neurochemical effects were inhibited by the administration of a CB1R antagonist (AM 251). Interestingly, a new generation of novel carboxamide synthetic cannabinoids have continued [61] to appear in the market as marijuana alternatives to evade drug control laws and cannabinoid blood/urine tests. For example, 5F-MDMB-PINACA (also known as 5F-ADB, 5F-ADB-PINACA), MDMB-CHIMICA, MDMB-FUBINACA, ADB-FUBINACA, and AMB-FUBINACA (also known as FUB-AMB and MMB-FUBINACA) were found to exhibit in vivo cannabinoid-like effects. Although all of these SCBs depressed locomotor activities, the time that is required for them to induce depressive activity varies. For example, 5F-MDMB-PINACA and MDMB-CHIMICA induced (30 min) depression of locomotor activity. ADB-FUBINACA and AMB-FUBINACA depressed locomotor activity for 60–90 min, whereas MDMB-FUBINACA depressed locomotor activity for 150 min. Furthermore, AMB-FUBINACA induced tremors at the highest dose tested. Furthermore, 5F-MDMB-PINACA, MDMB-CHIMICA, MDMB-FUBINACA, ADB-FUBINACA, and AMB-FUBINACA completely replaced the discriminative stimulus effects of Δ^9^-THC following 15-min preadministration. Together, these findings suggest that these SCBs may have abuse potential that is comparable to that of Δ^9^-THC. The AMB-FUBINACA may induce an increased risk of harmfulness in recreational users [61].

Abused SCBs have been shown to cause convulsion, but the mechanisms are not well understood. A recent study compared the convulsant effects of ∆^9^-THC, JWH-018, and 5F-AB-PINACA as well as that of a classic chemical convulsant pentylenetetrazol (PTZ) using an observational rating scale in mice [46]. JWH-018 and 5F-AB-PINACA elicited severe convulsions when compared to those elicited by PTZ, and ∆^9^-THC failed to elicit convulsions. SR141716A, but not PTZ, blocked the effects of JWH-018 and 5F-AB-PINACA. Nonselective inhibition of CYP450s with 1-aminobenzotriazole (1-ABT) potentiated the hypothermic effects of JWH-018 and 5F-AB-PINACA and provided protection against the convulsant effects of JWH-018 but not those of 5F-AB-PINACA or PTZ. These findings suggest that SCB-elicited convulsions are facilitated by high intrinsic efficacy at CB1Rs and indicate that drug metabolism plays an essential role in the in vivo efficacy of SCBs. Systemic administration of JWH-073, JWH-210, and Δ^9^-THC failed to affect sensorimotor gating, and locomotor activity was only partially affected. However, Δ^9^-THC, but not JWH-073 and JWH-210, induced an anxiolytic-like effect [51]. In vitro experiments have suggested that AB-PINACA displays a comparable affinity for CB1Rs but a greater efficacy for G-protein activation and a higher potency for adenylyl cyclase (AC) inhibition than Δ^9^-THC. Chronic administration of AB-PINACA also causes greater desensitization of CB1Rs (e.g., tolerance) than chronic administration of Δ^9^-THC. Notably, monohydroxy metabolites of AB-PINACA retain their affinity and full agonist activity at CB1Rs. Furthermore, the systemic administration of AB-PINACA and 4OH-AB-PINACA caused hypothermia in a CB1R-dependent manner [62]. These data demonstrate that AB-PINACA exhibits pronounced adverse effects through atypical pharmacodynamic properties at CB1Rs as compared to the effects of Δ^9^-THC by producing metabolically stable active phase I metabolites. These findings indicate that SCBs found in Spice products may exert their acute deleterious effect through CBR. However, a limited number of long-term studies have suggested that SCBs may induce severe harmful effects via complex neuroadaptation mechanisms, and future investigations on this topic are necessary.

In summary, investigation on the psychoactive components of marijuana, as well as the role of the eCB system in humans and its relationship to various brain disorders, has received much attention since the identification of CB receptors and their eCB ligands. Moreover, in addition to the well-known symptoms of euphoria and pleasure, SCBs binding to CB1R can also cause anxiety, short-term memory loss, and attention deficits and can have many other cognitive, affective, and psychomotor effects. The extent to which brain development and functions are disturbed remains unknown in young SCB users who have been shown to develop an increased risk of CB dependence. Although studies on the acute effects of SCBs are underway, studies on the chronic abuse of SCB drugs are still insufficient; thus, this area requires future attention. The immediate results of acute SCBs are probably related to the stimulation of presynaptic CB1R-mediated signaling cascades and the inhibition of the release of several neurotransmitters in the brain. In chronic SCB users, it can be presumed that adaptive changes in the CB1R-mediated signaling cascades and the related neurotransmitter systems result in severe adverse effects. It is likely that the additional compounds that were identified in the Spice/K2 preparations might also contribute, through CB1R or CB2R signaling, to the behavioral effects that are produced by smoking “Spice/K2”. In addition, their different pharmacological characteristics might cause the harmful effects of different preparations of “Spice/K2” to vary. Further investigation into these additional SCBs is required. “Spice/K2” is marketed as a “natural” herbal blend but contains at least one very potent SCB that acts through CB1R signaling, which likely accounts for the severe deleterious effects that are elicited by smoking “Spice/K2”.

## Figures and Tables

**Table 1:** The Classification of Cannabinoids (CBs) [32].

Class	Examples
Classical cannabinoids	Δ^9^-THC, HU-210, AM-906, AM-411, O-1184.
Non-classical cannabinoids	CP-47,497-C8, CP-55,940, CP-55,244.
Hybrid cannabinoids	AM-4030.
Aminoalkylindoles	JWH-018, JWH-073, JWH-398, JWH-015, JWH-122, JWH-210, JWH-081, JWH-200, WIN-55,212, JWH-250, JWH-251, Pravadoline, AM-694, RSC-4.
Eicosanoids	Anandamide and methanandamide.
Others	Diarylpyrazoles (SR141716A), naphtoylpyrroles (JWH-307), naphthylmethylindenes or derivatives of naphthalene-1-yl-(4-pentyloxynaphthalen-1-yl) methanone (CRA-13).

**Table 2:** Some known physiological effects of synthetic cannabinoids (SCBs) [33].

Raised heart rate & blood pressure
Altered state of consciousness
Mild euphoria and relaxation
Perceptual alterations (time distortion)
Intensification of sensory experiences
Pronounced cognitive effects
Impaired short-term memory
Agitation, seizures, hypertension, emesis and hypokalemia
Increase in reaction times

**Table 3:** Effects of SCBs identified in Spice/K products.

SPCs	Dose	Animal	Exposure	Parameter	Effects	Reference
JWH-018	3 mg/kg (i.p.)	Mouse	Acute	Cannabinoid tetrad	>Δ^9^-THC	[39]
	0.032 mg/kg (i.v.)	Monkey	Acute	Drug discrimination	>Δ^9^-THC	[40]
	3 mg/kg (i.p.)	Rat	Acute	Drug discrimination	>AM5983 >AM2233 >WIN 55, 212-2 >Δ^9^-THC	[41]
	2.5 mg/kg (i.p.)	Rat	Acute	Locomotion and catalepsy	>Δ^9^-THC	[42]
	5.8% (10–50 mg plant material), inhale	Mouse	Acute	Cannabinoid tetrad	>Δ^9^-THC	[43]
	0.1–1.8 μmol/kg (i.v.)	Mouse	Acute	Cannabinoid tetrad	>Δ^9^-THC	[44]
	0.03–0.3 mg/kg (i.p.)	Mouse	Acute	Cannabinoid tetrad	Induced	[45]
	10 mg/kg (i.p.)	Mice	Acute	convulsion	Induced	[46]
	2 and 3 mg/kg (vapor)	Human	Acute	neurocognitive function and subjective feelings	Impaired	[47]
	1 and 100 nM	HP Neuron	Acute	mEPSC frequency	Reduced	[48]
	5 nM–5 μM	Mouse brain slice	Acute	fEPSP	Impaired	[49]
JWH018 4-hydroxyindole metabolite	10 mg/kg (i.p.)	Mouse	Acute	Cannabinoid tetrad	>Δ^9^-THC	[39]
JWH-167	0.1–6.0 μmol/kg (i.v.)	Mouse	Acute	Cannabinoid tetrad	>Δ^9^-THC	[44]
JWH-203	0.13–13 μmol/kg (i.v.)	Mouse	Acute	Cannabinoid tetrad	>Δ^9^-THC	[44]
JWH-204	0.8–2 μmol/kg (i.v.)	Mouse	Acute	Cannabinoid tetrad	>Δ^9^-THC	[44]
JWH-205	13–19 μmol/kg (i.v.)	Mouse	Acute	Cannabinoid tetrad	>Δ^9^-THC	[44]
JWH-251	0.9–6 μmol/kg (i.v.)	Mouse	Acute	Cannabinoid tetrad	>Δ^9^-THC	[44]
JWH-208	2.8–38 μmol/kg (i.v.)	Mouse	Acute	Cannabinoid tetrad	>Δ^9^-THC	[44]
JWH-237	1.5–3.0 μmol/kg (i.v.)	Mouse	Acute	Cannabinoid tetrad	>Δ^9^-THC	[44]
JWH-306	1.1–2.9 μmol/kg (i.v.)	Mouse	Acute	Cannabinoid tetrad	>Δ^9^-THC	[44]
AM2389	0.1–0.3 mg/kg (i.p.)	Rat	Acute	Hypothermia and Drug discrimination	>AM5983 >Δ^9^-THC	[50]
AM5983	3 mg/kg (i.p.)	Rat	Acute	Drug discrimination	>JWH-018 >AM2233 >WIN 55, 212-2 >Δ^9^-THC	[41]
CP47,497	2.5 mg/kg	Rat	Acute	Locomotion and catalepsy	>JWH-018 >Δ^9^-THC	[42]
Cannabicyclo-hexanol	2.5 mg/kg	Rat	Acute	Locomotion and catalepsy	>CP47,497 >JWH-018 >Δ^9^-THC	[42]
JWH-073	3.2–32 mg/kg (i.v.)	Monkey	Acute	Drug discrimination	>Δ^9^-THC	[40]
	0.1–5 mg/kg	Rat	Acute	Locomotor activity, Anxiety and Sensorimotor gating	Reduced locomotor activity	[51]
JWH-210	0.1–5 mg/kg	Rat	Acute	Locomotor activity, Anxiety and Sensorimotor gating	Reduced locomotor activity	[51]
AB-001	0.3–30 mg/kg (i.p.)	Mouse	Acute	Hypothermia	> JWH-018 >Δ^9^-THC	[52]
JWH-081	0.625–1.25 mg/kg (i.p.)	mouse	Acute	LTP, Learning and memory	Impaired	[53]
AM-4054	0.1–1 mg/kg (s.c.)	Mouse	Chronic	Analgesia	Induced antinociception	[54]
AM-4054	0.01–0.16 mg/kg (i.p.)	Rat	Acute	Two-choice operant	Impaired	[55]
AM-7418	0.03-1 mg/kg (s.c.)	Mouse	Chronic	Analgesia	Induced antinociception	[54]
AM-411	0.32–1 mg/kg (i.m.)	Monkey	Acute and Chronic	Drug tolerance	>WIN 55,212-2 >Δ^9^-THC	[56]
AM-4054	0.0032–0.1 mg/kg (i.m.)	Monkey	Acute and Chronic	Drug tolerance	>AM-411 >WIN 55,212-2 >Δ^9^-THC	[56]
AM-2201	0.1–1 mg/kg (s.c.)	Rat	Acute	Hypothermia and Catalepsy	Induced	[57]
AM-2201	20 nM–2μM	Mouse brain slice	Acute	fEPSP	Impaired	[49]
UR-144	5.6 mg/kg (i.p.)	Mouse	Acute	Cannabinoid tetrad and Drug discrimination	>Δ^9^-THC	[58]
XLR-11	5.6 mg/kg (i.p.)	Mouse	Acute	Cannabinoid tetrad and Drug discrimination	=UR-144 >Δ^9^-THC	[59]
	20 nM–5μM	Mouse	Acute	fEPSP	Impaired	[49]
JWH-122	0.01–25 µM	Human	Endometrial stromal cell line	Stress and Cell death	Enhanced stress. No effect on cell death	[60]
5F-MDMB-PINACA	1.1 mg/kg (i.p.)	Rat	Acute	Locomotion	Reduced (30 min)	[61]
MDMB-CHIMICA	0.024 mg/kg (i.p.)	Rat	Acute	Locomotion	Reduced (30 min)	[61]
ADB-FUBINACA	0.19 mg/kg (i.p.)	Rat	Acute	Locomotion	Reduced (60–90 min)	[61]
AMB-FUBINACA	0.19 mg/kg (i.p.)	Rat	Acute	Locomotion	Reduced (60–90 min)	[61]
MDMB-FUBINACA	0.04 mg/kg (i.p.)	Rat	Acute	Locomotion	Reduced (150 min)	[61]
5F-AB-PINACA	10 mg/kg (i.p.)	Mice	Acute	Convulsion	Induced	[46]
AB-PINACA	1–10 mg/kg (i.p.)	Mice	Acute	Hypothermia	Induced	[62]
	0.2 mg/kg	Rat	Chronic	Learning and memory Locomotion Anxiety	Impaired Decreased Decreased	[63]
4-OH-AB-PINACA	30 and 10 mg/kg (i.p.)	Mice	Acute	Hypothermia	induced	[62]
5F-AMB	300 nM	Mice mPFC slices	Acute	Excitatory and inhibitory synaptic transmission	Impaired sEPSP, mEPSP, sIPSP and mIPSC	[64]
MMB-FUBINACA	Dose responses (i.p.)	Mice	Acute	Drug discrimination	Substituted for THC	[65]
CUMYL-PICA	Dose responses (i.p.)	Mice	Acute	Drug discrimination	Substituted for THC	[65]
5F-CUMYL-PICA	Dose responses (i.p.)	Mice	Acute	Drug discrimination	Substituted for THC	[65]
NNEI	Dose responses (i.p.)	Mice	Acute	Drug discrimination	Substituted for THC	[65]
MN-18	Dose responses (i.p.)	Mice	Acute	Drug discrimination	Substituted for THC	[65]
AB-FUBINACA	4.0 mg/kg (i.p.)	Rat	Chronic	Learning and memory Locomotion anxiety	Impaired Decreased Decreased	[66]
AB-CHMINACA	1.0 mg/kg (i.p.)	Rat	Chronic	Hypothermia Antinociception Anxiety Spatial memory depression	Induced No effect Reduced Impaired No effect	[66]
PB-22	0.4 mg/kg (i.p.)	Rat	Chronic	Hypothermia Antinociception Anxiety Spatial memory Depression	Induced No effect Reduced Impaired Induced	[66]
JWH-018 JWH-018-R	0.01–1 mg/kg (i.p.)	Mice	Acute	Locomotion Learning and memory LTP	Impaired Impaired Impaired	[67]
JWH018 Cl JWH-018 Br	0.01–1 mg/kg (i.p.)	Mice	Acute	Hypothermia Catalepsy Locomotion	Induced Induced Impaired	[68]
JWH-018	0.01–6 mg/kg (i.p.)	Mice	Acute	Convulsions Seizures Hyperreflexia Myoclonias Visual placing response Visual object response Acoustic Response Locomotion	Induced Induced Induced Induced Reduced Reduced Reduced Reduced	[69]
AKB48 5F-AKB48	0.01–6 mg/kg (i.p.)	Mice	Acute	Convulsions Hyperreflexia Myoclonias Catalepsy Hypothermia Immobility Acoustic response Visual placing response DA release	Induced Induced Induced Induced Induced Induced Reduced Reduced Increased	[70]
JWH-250 and JWH-073	0.01–15 mg/kg (i.p.)	Mice	Acute	Convulsions Hyperreflexia Myoclonias Aggressive responses Visual object response Visual placing response Hypothermia DA release	Induced Induced Induced Induced Induce Induced Reduced Increased	[71]
JWH018 AKB48	0.03–1 mg/kg (i.p.)	Mice	Acute	Locomotion DA release	Increased Increased	[72]
5F-ADBINACA AB FUBINACA STS-135	0.01–6 mg/kg (i.p.)	Mice	Acute	Hypothermia Catalepsy Locomotion Sensorimotor responses Pain threshold Seizures Myoclonia Hyperreflexia Aggressiveness	Induced Induced Reduced Reduced Increased Induced Induced Induced Increased	[73]

DA: Dopamine; HP: Hippocampus; EPSP: Excitatory postsynaptic potentials; fEPSP: field excitatory postsynaptic potential; mPFC: medial prefrontal cortex; IPSP: inhibitory postsynaptic potential; i.p.: Intraperitoneal; i.v.: intravenous; s.c.: Subcutaneous; i.m.: Intramuscular.
